# Epidemiology of maxillofacial injury among adults in sub-Saharan Africa: a scoping review

**DOI:** 10.1186/s40621-023-00470-5

**Published:** 2023-11-15

**Authors:** Adekunle I. Adeleke, Mbuzeleni Hlongwa, Sizwe Makhunga, Themba G. Ginindza

**Affiliations:** 1 Discipline of Public Health Medicine, School of Nursing and Public Health, University of KwaZulu-Natal, Mazisi Kunene Road, Durban, 4041 South Africa; 2 Public Health, Societies, and Belonging, Human Sciences Research Council, Pretoria, South Africa; 3 Cancer and Infectious Diseases Epidemiology Research Unit (CIDERU), College of Health Sciences, University of KwaZulu-Natal, Durban, South Africa

**Keywords:** Maxillofacial injury/facial trauma, Epidemiology, Risk factors, Cost, Sub-Saharan Africa

## Abstract

**Background:**

Injuries remain one of the leading causes of death globally. These disproportionately affect young adults and are particularly prevalent in sub-Saharan Africa (SSA). Maxillofacial injuries (MI) pose significant challenges to public health systems. However, much remains unknown regarding the epidemiology and extent of the financial burden in resource-limited areas, such as SSA, further necessitating more research and support. This scoping review aims to investigate the mechanism, distribution, and financial impact of MI in adults aged ≥ 18 years in SSA.

**Main body:**

The scoping review was guided by the methodological frameworks of Arksey and O’Malley and Levac. An electronic literature search for English-published articles on maxillofacial injuries in adults ≥ 18 years was conducted in Scopus, Medline, PubMed, Science Direct, CINAHL, Health Source: Nursing/Academic Edition, and grey literature. The PRISMA chart was used to document database searches and screening outcomes while reporting was guided by PRISMA-ScR. The data extraction process revolved around the predefined study outcomes, which encompassed the study characteristics and epidemiological parameters. The review used a narrative approach to report findings and evaluate publication quality using the STROBE checklist.

The database search yielded 8246 studies, of which 30 met the inclusion criteria. A total of 7317 participants were included, 79.3% of whom were males. The peak age range for incidence was between 18 and 40 years. Road traffic collision (RTC) was the leading cause of MI, 59% of which resulted from motorcycle collisions. Assault/interpersonal violence ranked as the second leading cause of MI. The mandible was MI’s most frequently affected hard tissue, followed by the midface. Factors such as alcohol/illicit drug use, poor knowledge of traffic regulations, and non-observance of these regulations were associated with MI. In our study, the cost range for mandibular fractures was $200-$468.6, borne by victims and their families.

**Conclusions:**

Maxillofacial injuries are predominantly caused by road traffic collisions and assaults in SSA. The findings can provide valuable insights into policy decisions and prevention strategies aimed at reducing injury burden. Further research is warranted to explore the psychological impact of MI, including PTSD, for tailored support and intervention.

*Scoping Review Registration* The protocol has been registered on the Open Science Framework. *Registration* DOI: https://doi.org/10.17605/OSF.IO/BWVDK.

**Supplementary Information:**

The online version contains supplementary material available at 10.1186/s40621-023-00470-5.

## Background

Globally, injuries remain one of the significant causes of death annually (Ritchie et al. 2018; Heron 2017), persisting at a consistent rate of 7.3% of global Disability-Adjusted Life Years (DALYs) between 1990 and 2019 (Vos et al. 2020). These account for nearly one in 10 deaths of 15- to 49-year-olds worldwide, with mortality rates varying across countries (Ritchie et al. 2018). Studies have revealed that approximately 89% of deaths attributable to injuries are from low- and middle-income countries (LMICs), whereas 10% are from sub-Saharan Africa (SSA) (Heron 2017; Norton and Kobusingye 2013). Hence, the burden of injuries in SSA is projected to remain significant over the next 20 years (Norton and Kobusingye 2013). The facial region has been described as the most exposed part of the body, fragile in structure, and lacking protection (VandeGriend et al. 2015; Saperi et al. 2017), making it a prime cause of morbidity (Wu et al. 2021). The National Trauma Data Bank report of 2016 revealed that approximately 25% of all injuries involve the face (Choi et al. 2020), with a global incidence of approximately 8 million (Wu et al. 2021). The western region of SSA observed a remarkable 117% increase in the incidence of facial fractures between 1990 and 2017 (Lalloo 2017). Nevertheless, surveillance systems for injuries in this region may encounter distinct challenges that can impede accurate assessment of the epidemiology of MI. Studies conducted in Europe and Asia have identified MI as a prevalent condition in maxillofacial surgery services (Juncar et al. 2021; Manodh et al. 2016; Boffano et al. 2015); therefore, the cost of treatment and potentially irreversible damage pose a challenge to public health services (Lalloo 2017; Abosadegh and Rahman 2018).

Trauma to the maxillofacial region may present as burns, lacerations, blunt traumatic injuries to the soft tissues, or fractures of the facial bones (nasal, maxilla, zygoma, and mandible (Alqahtani et al. 2020; Boonkasem et al. 2015). Published studies have revealed the mechanism of this nature of injury to be related to road traffic collision (RTC) (Xiao-Dong et al. 2020; Agbara et al. 2021), assaults (Adeyemo et al. 2005; Boyes and Fan 2020), falls (Al-Bokhamseen and Al-Bodbaij 2019; Brucoli et al. 2020), contact sports (Secanho et al. 2021; Diab et al. 2021), animal attacks (Juncar et al. 2021; Ghezta et al. 2019), and occupational injuries (Roccia et al. 2022; Goedecke et al. 2019). Studies from Asia (Alqahtani et al. 2020; Xiao-Dong et al. 2020) and South America (Aires et al. 2020; Ferreira Lima de Moura et al. 2016) have revealed that the most common aetiology of maxillofacial fractures is RTC, followed by violence (Alqahtani et al. 2020; Xiao-Dong et al. 2020; Ferreira Lima de Moura et al. 2016). Recent research from Australia and Europe indicates that incidents of assault are on the rise, surpassing those attributed to RTC (Diab et al. 2021; Diab et al. 2022; Shumynskyi et al. 2022), and physical aggression has been identified as a significant aetiology of this trauma (Santos et al. 2018; Pillay et al. 2018; Conceição et al. 2018). Several published studies have demonstrated considerable differences in the demographic characteristics of MI between female and male patients, with differences in age distribution, aetiology, and associated injuries (Alqahtani et al. 2020; Conceição et al. 2018; Wusiman et al. 2020). Domestic abuse has been reported to be an essential driver of facial fractures in females (Lalloo 2017) and can be used as a marker for attempted femicide (Mayrink et al. 2020). Other factors include the type of accident (RTC), interpersonal violence (Ribeiro et al. 2016), and habit and social factors that change from adolescence to adulthood (Ferreira et al. 2014). In addition, unemployment (Santos et al. 2018), low socioeconomic status (Kruger and Tennant 2016), and substance use (Sorenson et al. 2021; Yarmohammadi et al. 2020) have been identified as risk factors. Globally, alcohol use has been identified as a leading risk factor for facial injury for 25–49 years (Conceição et al. 2018; Bhandari et al. 2019; Murray et al. 2020).

Facial trauma has profound effects on individuals, negatively influencing their social and emotional well-being and potentially causing social exclusion (Ferreira Lima de Moura et al. 2016). It can also impact work performance, increase absenteeism, and increase the risk of job loss (Lamoglia and Minayo 2009). Hence, creating significant socioeconomic challenges and increasing the need for social services (Ferreira Lima de Moura et al. 2016; Lamoglia and Minayo 2009). Facial trauma can be disabling, resulting in simple to complex surgical care and rehabilitation to restore aesthetic, physical, and functional damage (Saperi et al. 2017; Brucoli et al. 2020; Pena et al. 2014). However, restoring injured tissues may require an interdisciplinary approach to minimize the long-term negative effects (Chukwulebe and Hogrefe 2019). Facial fracture treatment is expensive. In high-resource countries, the cost of mandibular fracture treatment ranges from $26,000 to $50,000 in the US (Nalliah et al. 2013), and from $793 to $12,780 in Australia (Moncrieff et al. 2004). In LMIC, Malaysia, the treatment costs are between $1,261 and $1,716 (Saperi et al. 2017). In resource-limited areas such as SSA, Nigeria’s treatment costs an average of $488 (Akhiwu et al. 2015) and places a heavy financial burden on patients due to the lack of insurance and social protection (Akhiwu et al. 2015; Famurewa et al. 2021). Inadequate injury data in this region hinders policy development, resource allocation, and targeted injury prevention interventions. Analysing diverse behavioural patterns in SSA countries can offer insights into the prevention of facial injuries. Lastly, owing to limited local injury data, weak injury surveillance systems, and high MI costs, this study aims to explore MI mechanisms, distribution, and financial impact in adults aged ≥18 years in SSA.

This scoping review systematically mapped available research focused on the epidemiology of MI among adults in SSA to summarize the evidence and identify gaps.

## Main text

### Methods

The protocol for this scoping review has been published elsewhere (Adeleke et al. 2023). A scoping review maps the literature on a topic by identifying key concepts, theories, and sources of evidence that inform practice in the field (Arksey and O’Malley 2005). This scoping review employed the available literature (peer-reviewed and grey) on the distribution of MI involving adults in SSA underpinning, prevalence, incidence, risk factors, mortality, and economic burden. Database searches and screening outcomes from diverse studies have been reported using the preferred reporting items for systematic reviews and meta-analysis (PRISMA) flow diagram. The methodological frameworks described by Arksey and O’Malley (Arksey and O’Malley 2005) and Levac’s methodological enhancement for scoping review projects (Levac et al. 2010) were used for guiding this review. The reporting was guided by the preferred reporting items for systematic reviews and meta-analysis extended for scoping reviews (PRISMA-ScR) checklist and explanation (Tricco et al. 2018) included in Additional File 1 [PRISMA-ScR Checklist].

#### Step 1: Research questions

The eligibility for the research question was determined using the population, concept, and context (PCC) framework (Peters et al. 2017), as shown in Table 1.

**Table 1:** PCC framework for defining the eligibility of the studies for the principal research question

Population	Adults, 18 Years and above with Maxillofacial Injury
Concept	Maxillofacial injury
Context	Countries in sub-Saharan Africa

Based on the PCC framework, the following research questions were proposed:

#### Principal research question

What is the existing evidence regarding the characteristics and trends of maxillofacial injuries among adults in SSA?

#### Sub-questions

- What is the burden of maxillofacial injury in SSA, with estimations of the prevalence, incidence, and mortality?
- What risk factors are associated with maxillofacial injury in SSA?
- What are the estimated costs associated with maxillofacial injuries in SSA?

#### Step 2: Search strategy

A literature search was conducted from June to August 2022, on studies published in English. It identified the epidemiology of MI and the associated costs among adults aged 18 years and above in SSA. The authors, with the assistance of the Institution (UKZN) librarian performed an electronic literature search without a date limit, using the following databases: Scopus, Medline, PubMed, Science Direct, Cumulative Index to Nursing and Allied Health Literature (CINAHL), and Health Source: Nursing/Academic Edition. Furthermore, the authors searched grey literature from institutional repositories, government, and international organizations’ reports, such as the WHO, and from university dissertations and theses. To ensure comprehensive coverage and accuracy, searches were undertaken using Medical Subject Headings (MeSH) or subject headings search terms that relate to key concepts, as well as Boolean search terms “AND” and “OR.” The keyword terms used for the literature search included maxillofacial trauma, maxillofacial injury, facial trauma, facial injury, facial bone trauma, facial bone injury, facial lacerations, mandibular fracture, mandibular trauma, mandibular injury, maxillary fracture, maxillary injury, nasal fracture, nasal injury, orbital fracture, orbital injury, epidemiology, prevalence, incidence, risk factors, disability, mortality, burden, comorbidities, associated costs, and countries in sub-Saharan Africa. Furthermore, relevant articles were obtained from the reference list of the included articles (snowball approach). The details of the strategy employed for the literature search are presented in Additional File 2 [DATA BASE- Search].

#### Step 3: Study selection

Literature selection was based on the inclusion and exclusion criteria, which were developed by the principal investigator and screened by the other members of the research team.

Inclusion criteria

All full-text studies conducted in SSA among the adult population aged 18 years and above that presented evidence on any of the following criteria were eligible and considered:

- The incidence of Maxillofacial injury
- The Prevalence of Maxillofacial injury
- Distribution of maxillofacial injury
- Aetiology/risk factors of Maxillofacial injury
- The comorbidities associated the maxillofacial injury.
- Financial burden of Maxillofacial injury

Exclusion criteria were:

- Evident articles involving individuals under the age of 18 years.
- Review studies
- Studies conducted outside the setting of SSA.
- Clinical trials and intervention-based studies
- Studies conducted in languages other than English and those that did not have an English version.
- Studies that lack a clear definition of maxillofacial injury.

The screening process for articles was carried out in three stages: title, abstract, and full article screening. The compilation of relevant articles and deduplication of articles was achieved by employing the Endnote reference manager. Two reviewers were responsible for title and abstract screening to minimize the risk of selection bias. Full article screening was performed by two independent reviewers, and the attention of a third reviewer was sought for adjudication when there was a disagreement of opinion between the two reviewers. The full texts of potential articles were reviewed and evaluated against eligibility criteria.

#### Step 4: Charting data

Using Google Form (Additional File 3 [Extraction form]), the first author developed a data charting (extraction) form iteratively. The feedback obtained from the two pilot studies involving the data extraction form played a crucial role in ensuring its accuracy and facilitating the necessary adjustments made by the research team. The details captured were: (i) author and date of publication, (ii) study setting, (iii) publication type, (iv) study design (sample size), (v) peak age range, (vi) aetiology/mechanism of injury, (vii) affected tissues (soft and hard), (viii) cost, and (ix) other relevant findings.

#### Step 5: Collating, summarizing, and reporting results

The review’s primary outcome was the aetiology/mechanism of MI, peak age range of incidence, affected facial tissues (soft and hard), participant sex distribution, and financial impact. One reviewer (A.A) documented study details that include country of study, publication year, peak age range of incidence, sex, injury aetiology, mortality, and financial impact. No authors have been contacted to obtain any additional data. Post-literature review, weighted percentages were calculated for sex distribution, the peak age range of incidence, aetiology/mechanism of injury, and the distribution of affected tissues (hard and soft). The financial implications were presented within a range.

### Quality assessment

The strength of the included studies was determined by the Strengthening the Reporting of Observational Studies in Epidemiology (STROBE) statement (Elm et al. 2014). Considering our included observational studies, the STROBE tool proved appropriate for ensuring quality, credibility, and comprehensive and transparent reporting in our review. Two independent reviewers conducted all assessments. The STROBE checklist comprised 22 items, distributed as one for the abstract, two for the introduction, nine for the methods, five for the results, four for the discussion, and one for funding. Disagreements were resolved through consensus.

## Results

**Figure 1:**
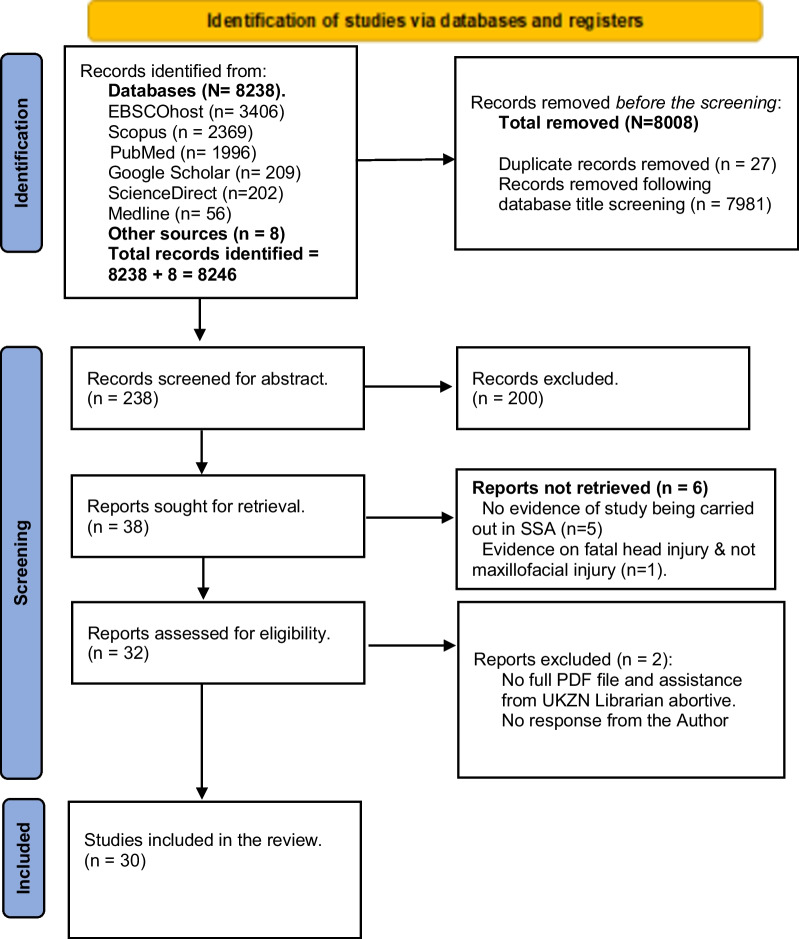
PRISMA 2020 flow diagram for articles screening and selection

An electronic search identified 8246 articles. After title screening, 7981 and 27 articles were excluded due to irrelevance and duplicate publication, respectively. Two hundred and thirty-eight articles were screened for abstracts, and 38 articles were eventually retrieved. Thirty (30) studies met the inclusion criteria and were included in the scoping review. The details of the selection process are presented in the flowchart in Figure 1.

A narrative approach was adopted to report the findings of the scoping review. This approach was primarily owing to the heterogeneity of the methodologies adopted in the included studies. Most studies lacked uniformity in the assessment and measurement of variables. Among these variables is the age bracket of peak incidence, which differs from one author to another. Some studies employed the mean age (Famurewa et al. 2021; Stanford-Moore et al. 2022; Tekin and Ali 2021), others used the age bracket (Agbara et al. 2021; Pillay et al. 2018; Akhiwu et al. 2015; Tugaineyo 2011; Tsakiris et al. 2002; Obimakinde et al. 2018; Agbor et al. 2014; Mogajane and Mabongo 2018; Kuye and Olufemi 2022; Chalya et al. 2011; Mpiima et al. 2018; Oginni et al. 2016; Sohal et al. 2019; Obimakinde et al. 2017; Krishnan et al. 2017; Bernard et al. 2012; Kiprop 2019; Nyameino et al. 2018; Moshy et al. 2020; Nwashindi et al. 2015; Kileo 2012; Stanslaus 2017; Majambo et al. 2013; Udeabor et al. 2012; Kamulegeya et al. 2009), and others adopted the combination of the two (Agbara et al. 2018; Teshome et al. 2017). Likewise, one of the included study authors restricted the findings to soft tissues (Bernard et al. 2012), some to hard tissues (Akhiwu et al. 2015; Famurewa et al. 2021; Tsakiris et al. 2002; Obimakinde et al. 2018; Mogajane and Mabongo 2018; Kuye and Olufemi 2022; Mpiima et al. 2018; Oginni et al. 2016; Obimakinde et al. 2017; Krishnan et al. 2017; Kiprop 2019; Moshy et al. 2020; Nwashindi et al. 2015; Kamulegeya et al. 2009), while others adopted a combination of soft and hard tissues (Agbara et al. 2021; Pillay et al. 2018; Stanford-Moore et al. 2022; Tekin and Ali 2021; Tugaineyo 2011; Agbor et al. 2014; Chalya et al. 2011; Sohal et al. 2019; Nyameino et al. 2018; Kileo 2012; Stanslaus 2017; Majambo et al. 2013; Udeabor et al. 2012; Agbara et al. 2018; Teshome et al. 2017). Some studies were limited to the mandible (Akhiwu et al. 2015; Famurewa et al. 2021; Mpiima et al. 2018; Moshy et al. 2020; Stanslaus 2017), some to the hard tissue of the middle third of the face (Krishnan et al. 2017; Udeabor et al. 2012), and some to generalized facial bones (Agbara et al. 2021; Pillay et al. 2018; Stanford-Moore et al. 2022; Tekin and Ali 2021; Tugaineyo 2011; Tsakiris et al. 2002; Obimakinde et al. 2018; Agbor et al. 2014; Mogajane and Mabongo 2018; Kuye and Olufemi 2022; Chalya et al. 2011; Oginni et al. 2016; Sohal et al. 2019; Obimakinde et al. 2017; Kiprop 2019; Nyameino et al. 2018; Nwashindi et al. 2015; Kileo 2012; Majambo et al. 2013; Kamulegeya et al. 2009; Agbara et al. 2018; Teshome et al. 2017). In contrast, the financial obligation required in the management was limited to mandibular fracture treatment (Akhiwu et al. 2015; Famurewa et al. 2021; Agbor et al. 2014).

The reviewed 30 articles (full text) comprised 16 cross-sectional studies (Akhiwu et al. 2015; Tekin and Ali 2021; Agbor et al. 2014; Mogajane and Mabongo 2018; Chalya et al. 2011; Mpiima et al. 2018; Sohal et al. 2019; Bernard et al. 2012; Nyameino et al. 2018; Moshy et al. 2020; Kileo 2012; Stanslaus 2017; Majambo et al. 2013; Udeabor et al. 2012; Kamulegeya et al. 2009; Agbara et al. 2018), 12 retrospective studies (Agbara et al. 2021; Pillay et al. 2018; Famurewa et al. 2021; Tugaineyo 2011; Tsakiris et al. 2002; Obimakinde et al. 2018; Oginni et al. 2016; Obimakinde et al. 2017; Krishnan et al. 2017; Kiprop 2019; Nwashindi et al. 2015; Teshome et al. 2017), and two cohort studies (Stanford-Moore et al. 2022; Kuye and Olufemi 2022). The findings were sectioned under the following themes: distribution and the characteristics of included studies, sex distribution of the participants, the peak age range of incidence, aetiological factors, soft tissue affected, pattern of fracture, burden of disease, and financial burden.

### Distribution and the characteristics of included studies

A significant part of the included studies were conducted in Nigeria with ten articles (33.3%) (Agbara et al. 2021; Akhiwu et al. 2015; Famurewa et al. 2021; Obimakinde et al. 2018; Kuye and Olufemi 2022; Oginni et al. 2016; Obimakinde et al. 2017; Nwashindi et al. 2015; Udeabor et al. 2012; Agbara et al. 2018), followed by Tanzania with five articles (16.7%) (Chalya et al. 2011; Sohal et al. 2019; Moshy et al. 2020; Kileo 2012; Stanslaus 2017). Kenya was with four articles (13.3%) (Tugaineyo 2011; Bernard et al. 2012; Kiprop 2019; Nyameino et al. 2018), while South Africa (Pillay et al. 2018; Tsakiris et al. 2002; Mogajane and Mabongo 2018) and Uganda (Mpiima et al. 2018; Krishnan et al. 2017; Kamulegeya et al. 2009) were of three articles each (10.0%). Rwanda had two articles (6.7%) (Stanford-Moore et al. 2022; Majambo et al. 2013), whereas Somalia (Tekin and Ali 2021), Cameroon (Agbor et al. 2014), and Ethiopia (Teshome et al. 2017) had one article each (3.3%) (Table 2).

**Table 2.** Authors’ names, STROBE score, location of study, gender distribution, and aetiology of injury

Author and date	STROBE Score	Country of study	Male/female ratio	The major cause of injury (%)	2nd major cause of injury	Interpersonal violence (%)
Agbara et al. (2018)	20	Nigeria	5.2:1	Road traffic crashes (86.5%). Vehicular crashes = 55%, Motorcycle crashes = 44.6%	Assault (5.3%)	N/A
Agbara et al. (2021)	20	Nigeria	8.8:1	Road traffic-related accident (74.2), 50.9% of this = motorcycle	Assault (N/A)	N/A
Agbor et al. (2014)	17	Cameroon	1.8:1	Only commercial motorcycle accident	Not specified	N/A
Akhiwu et al. (2015)	13	Nigeria	3.2:1	RTC only. Motorcycle related (54). Motor vehicle related (38)	Not specified	N/A
Bernard et al. (2012)	16	Kenya	3.3:1	Motor vehicle accidents (44.6)	Interpersonal violence (39.1)	39.1
Chalya et al. (2011)	15	Tanzania	2.7:1	Road Traffic Crash (57.1)	Assault (16.2)	16.8
Kiprop (2019)	21	Kenya	7.2:1	Interpersonal Violence (42.5)	Road Traffic Collision (40.1)	42.5
Famurewa et al. 2021	18	Nigeria	7.3:1	Road Traffic Collision (83). Bike = 40%	Assault (12)	12
Kamulegeya et al. (2009)	19	Uganda	7.7:1	Road traffic collision: (56.06%)	Assault (34.84%)	N/A
Kileo (2012)	20	Tanzania	8.8:1	Road Traffic Accident (64.2). Motorcycle = 53.4%	Assault (19)	N/A
Krishnan et al. (2017)	18	Uganda	12:1	Road Traffic Accident (49.1)	Assault (N/A)	N/A
Kuye and Olufemi (2022)	19	Nigeria	3:1	RTA (66). (Motor bike= 41), Vehicle accident (= 25)	Assault (20)	N/A
Majambo et al. (2013)	17	Rwanda	2.2:1	Road Traffic accident (59.9). Motorcycle = 24.7, motor vehicle = 20.9	Fall (17.6)	Assault (7.7)
Mogajane et al. (2018)	17	South Africa	4.5:1	Assault (60.3)	Motor vehicle accident (17.5)	N/A
Moshy et al. (2020)	17	Tanzania	10:1	Motorcycle crash (100)	N/A	N/A
Mpiima et al. (2018)	18	Uganda	7.7:1	Road traffic accident (58)	Assault (38)	N/A
Nwashindi et al. (2015)	16	Nigeria	2:1	Road Traffic Accident (80)	Fall (9%)	Assault (6)
Nyameino et al. (2018)	16	Kenya	5:1	Road Traffic Accident (motorcycling)	N/A	N/A
Obimakinde et al. (2017)	18	Nigeria	3.4:1	Road traffic collision (78.5). Motorcycle = 54.5	Assault (19.7)	N/A
Obimakinde et al. (2018)	19	Nigeria	4:1	Motorcycle collision (100)	N/A	N/A
Oginni et al. (2016)	19	Nigeria	4.4:1	Road Traffic crashes (86.1%). Motorcycle crashes (67.5%)	Fall (N/A)	N/A
Teshome et al. (2017)	20	Ethiopia	4.02:1	Interpersonal violence (75.8)	Road traffic collision (21.5%)	N/A
Tsakiris et al. (2002)	17	South Africa	M>F, no specifics	A gunshot wound (100)	N/A	N/A
Stanslaus (2017)	16	Tanzania	6.7:1	Motorcycle accident (100)	N/A	N/A
Pillay et al. (2018)	15	South Africa	2.6:1	Interpersonal violence (55%)	Road Traffic Accidents (16%)	N/A
Sohal et al. (2019)	18	Tanzania	37.7:1	RTA (Motorcycle)	N/A	N/A
Stanford-Moore et al. (2022)	14	Rwanda	16.9:1	Road Traffic Accident (40.71). Motorcycle = 33.3	Assault (29.6)	N/A
Tekin and Ali 2021	20	Somalia	5:1	Interpersonal violence (71.2), of which the majority was an explosion (24.4%) and assault (24.4%)	Sports accident (15.6%)	71.2
Tugaineyo (2011)	20	Kenya	4.6:1	Road Traffic injury (61), motorcycle accident =31% followed by Motor vehicle accident = 22%	Interpersonal violence (27.6)	27.6
Udeabor et al. (2012)	15	Nigeria	5.3:1	Road traffic accident (91.1), Motorcycle = 45.5%	Assault (4%)	N/A

Twenty (66.7%) of the included articles focused on facial injury in general (Pillay et al. 2018; Stanford-Moore et al. 2022; Tekin and Ali 2021; Tugaineyo 2011; Tsakiris et al. 2002; Obimakinde et al. 2018; Mogajane and Mabongo 2018; Kuye and Olufemi 2022; Chalya et al. 2011; Oginni et al. 2016; Sohal et al. 2019; Obimakinde et al. 2017; Krishnan et al. 2017; Nyameino et al. 2018; Nwashindi et al. 2015; Kileo 2012; Majambo et al. 2013; Kamulegeya et al. 2009; Agbara et al. 2018; Teshome et al. 2017), whereas five (16.7%) specifically explored mandibular fractures (Agbara et al. 2021; Mpiima et al. 2018; Kiprop 2019; Moshy et al. 2020; Stanslaus 2017). Three (10.0%) articles (Akhiwu et al. 2015; Famurewa et al. 2021; Agbor et al. 2014) were on the cost of management, one (3.3%) article was on soft tissue injury of the face (Bernard et al. 2012), and one (3.3%) article was on the middle third of the face (Udeabor et al. 2012). The findings of these studies are summarized in Tables 2 and 3. The STROBE checklist was used to determine the publication strength of the included articles. The average STROBE score for the included studies was 17.6 (Additional File 4 [STROBE Score for included studies]), signifying the quality, transparency, and completeness of the included studies.

**Table 3:** Summary characteristics of the included studies

Author & date	Publication Type	Study design (Sample size)	The peak age range of incidence, year (%)	Maxillofacial soft tissue affected (%)	Most maxillofacial bone affected (%)	2nd Most Maxillofacial bone affected (%)	Cost of Management ($)	Relevant findings
Udeabor et al. (2012)	Journal article	Cross-sectional (303)	Third decade—20–29 (–)Mean age = 28.8	Frontal (11.96) Lips (6.7)	Middle third (24.9) and the zygomatic complex was = 9.74%	Mandible (10.23)	N/A	Only 6.9% of the victims in vehicular crashes wore the seat belt. Only 1.7% of those involved in motorcycle crashes wore head helmets. GCS ≤8 to 15. Complications include malocclusion and infection
Adeyemo et al. (2005)	Journal article	Retrospective (519)	20–39 (58.8)	Lip (2.2), frontal (2.5) and cheek (2.0)	Mandible (52.1)	Zygomatic complex (12)	N/A	Factors such as age and sex were associated with facial fractures. The body and parasymphyseal regions appear to be the commonest sites affected in the mandible
Obimakinde et al. (2018)	Journal article	Cross-sectional (387)	21–30 (39.8)	General (91.2)	Mandible (45.3)	Maxilla (25.6)	200–240 (Direct cost for the procedure only)	Trauma to the teeth = 323 (83.5%) Loss of tooth from 62.3% of participants.12.3% and 51.5% of participants perceived that the treatment was expensive. Incidence is greater in the urban area. The prevention will serve as a form of poverty reduction as money expended by the victim in the treatment of injuries will serve to better their economic status
Tugaineyo (2011)	Journal article	Cross-sectional (50)	21–30 (58)	N/A	Mandible (N/A)	N/A	488 (Direct and Indirect cost)	Furthermore, this equals 15.2 % of the GDP per capita of the year of study. The need for policies to be directed at imposing the use of safety gadgets
Krishnan et al. (2017)	Journal article	Cross-sectional (422)	26–35 (–)	General (81.7)	Mandible (50.4)	N/A	N/A	Incidence= 32.7% of patients from the A&E department. Alcohol consumption is associated with facial injury. Associated injuries are majorly head injuries and long bone fractures. Complications include malocclusion, infection, and hypertrophic scar
Kuye and Olufemi (2022)	Journal article	Cross-sectional (154)	21–30 (N/A)	92.2 (70.4)	Mandible (70.4)	Nasal fracture (11.1)	N/A	The use of alcohol use before injury was reported in about half (49.4%) of the population. Associated injury= head injury. ISS ≥ 16 (37.5% of the patients). Time of injury= Night. The mortality rate of 11.7%. GCS= 3-15. Complications = Infection and malocclusion
Bernard et al. (2012)	Dissertation	Retrospective (534)	20–29 (38.2)	N/A	Mandibular fracture (56.1), with the body recording the highest occurrence (28.2%)	Maxillary (14.2)	N/A	The highest incidence occurs on Saturdays and Sundays. One-third of the patients were on alcohol/substance use before sustaining of injury. Incidence was more in the urban area, at night. Associated injury =traumatic brain injury, Complication: malocclusion, infection, and Malunion
Moncrieff et al. (2004)	Journal article	Retrospective (100)	Mean 38.8	N/A	Mandible-Body (43)	Mandible-Parasymphysis (34)	468.61 (investigation, med.& hospitalize)	All patients from the low-income class paid out of pocket. Sixty-two per cent of the high-income class received the open reduction inter-maxillary fixation (ORIF) a more expensive procedure than the close reduction inter-maxillary fixation (IMF)
Majambo et al. (2013)	Journal article	Prospective cross-sectional (132)	21–30 (51.51%)	N/A	Mandible (68.94)	Midface (20.45) and maxilla were 30.1% of the midface	N/A	Associated injury: long bone (femoral) fracture =45.45% and skull = 18.18%. Head injury (Loss of consciousness) in 35.6%. Post-op complications= Infection (48.7%) and malocclusion (18%)
Nwashindi et al. (2015)	Dissertation	Cross-sectional (137)	21–30 (43)	Laceration (26.9)	Mandibular (45.2)	Midfacial (28)	N/A	Maxillofacial injuries are of higher occurrence in patients with low education levels, low social economic class, and urban residents. Complication: infection and malocclusion
Obimakinde et al. (2017)	Journal article	Retrospective (387)	18–27 (47.5)	N/A	Frontal (23)	Maxilla and Zygoma (8)	N/A	The study further emphasizes the need to enforce the use of protective gears
Mogajane and Mabongo (2018)	Journal article	Cohort (140)	26–35 (64.3)	N/A	Mandible (48)	Zygomatic bone And complex (28.1)	N/A	The classification of the causes of road accidents in Nigeria into human, mechanical, and environmental factors. The peak period of road crashes in June, the rainy season resulting in poor visibility and road wetness. Likewise, it is the “ember” period that is characterized by festivities with increased activities.
Ferreira Lima de Moura et al. (2016)	Journal article	Cross-sectional (182)	23–30 (53.8)	Lip (38.7)	Dentoalveolar Fracture (59.3)	Mandible (19.8)	N/A	Prevalence = 16% among patients that visited the clinic, particular attention to motorcycle accidents
Agbor et al. (2014)	Journal article	Cross-sectional (194)	20–39 (75)	N/A	Mandible (73)	Middle third (19) Zygoma (28.1% of the middle third	N/A	Most cases happened in the evenings and at night. Policy in ensuring that the rule of law has no tolerance for assault/violence
Nyameino et al. (2018)	Journal article	Cross-sectional (132)	21–40 (76.5)	N/A	Symphysis (37.9)	Parasymphyseal (29.5) and alveolar process (29.5)	N/A	About one-third of the injured were under the influence of alcohol. Most of the crashes occur at night (63, 47.7%), and victims were traveling at speeds between 31–60km/hour. The severity of the injury was associated with the type of helmet worn. The use of a half-face helmet increases the risk of facial fractures.
Chalya et al. (2011)	Journal article	Cross-sectional (73)	21–30 (48)	N/A	Mandibular and the majority (91%) were bilateral	N/A	N/A	Assault victims were under the influence of alcohol. The majority are of tertiary educational status and urban dwellers. The time of injury for assault victims was at night
Moshy et al. (2020)	Journal article	Retrospective (215)	21–40 (40)	N/A	Mandible (66)	Zygoma (34) of maxillary fracture	N/A	An increase in socioeconomic activities results in an increase in human and vehicular activities and an increase in motor traffic accidents, especially in the absence of corresponding stringent civil and traffic rules and regulations
Kiprop (2019)	Journal article	Cross-sectional (91)	21–30 (41.8)	98% in total. Upper lip (30.8)	Maxilla (92.3) orbital (33)	Mandible (20.9)	N/A	Most riders had neither formal training nor a riding license. Alcohol consumption by some of the victims
Sohal et al. (2019)	Journal article	Retrospective (233)	20–30 (N/A)	N/A	Mandible (68.2), with the body being the most frequent (25%)	Maxilla (31.8), Midface, zygomatic bone most frequent (29%)	N/A	Motorcycle riders were more involved in related injuries than pillion passengers. Most treatments are out of pocket; therefore, most patients opt for MMF and wire suspension which are cheaper. Concomitant injury = Head with GCS ≤15.
Tsakiris et al. (2002)	Journal article	Retrospective (151)	20–29 (41.4)	N/A	Mandible with the body being the most frequent (25)	The midface, with zygoma being most frequent (32.5%)	N/A	Above half of the patients (54.9) suffered an altered state of unconsciousness, a larger proportion of commuters do not wear crash elements
Mpiima et al. (2018)	Journal article	Retrospective (311)	21–30 (46)	N/A	Mandible (69.1) and 32.2% of this is the parasymphyseal fracture.	Midface (45.3%), and 34.7% of this is the zygomatic bone	N/A	More than a third (43.1%) of the patients presented with associated altered levels of consciousness, with a Glasgow coma scale of less than 15. Complication: Trismus and scar
Kamulegeya et al. (2009)	Journal article	Retrospective (326)	21–30 (47.2). Mean age= 29.1	49.4%	Mandible (75)	Maxillary (20.2)	N/A	Incidence is high in rural residences and associated with the use of alcohol. Concomitant injury (Head and neck) = 31.65%
Tekin and Ali (2021)	Journal article	Retrospective (211)	20–29 (N/A)	N/A	Mandible (61)	Maxilla (21)	N/A	Complications: Threatened airway (that may result in neural deficit and death) especially when the tongue, floor of the mouth, and bilaterally facial skeletal bone are affected. The incidence was majorly at night
Kileo (2012)	Dissertation	Cross-sectional (178)	21–30 (55.1)	General. Laceration (48.3)	Mandible (Symphysis) 36.5%	Mandible (Parasymphysis) 30.5%	N/A	Complications: A significant possibility of airway obstruction when the tongue, floor of the mouth, and bilaterally facial skeletal bone are affected. More incidence at night
Santos et al. (2018)	Journal article	Retrospective (239)	18–24 (27)	Lacerations (41.9)	Mandible (53.8)	Zygomatic bone (10.3)	N/A	Prevalence = 2.89%. There is a need for more training on maxillofacial imaging by Dentists and Medical doctors on diagnostic imaging to avoid the chances of fractures being misdiagnosed
Oginni et al. (2016)	Journal article	Cross-sectional (116)	20–39 (81)	Orbit/Ocular region (61.5%)	Mandible (71.2), parasymphyseal fracture (41.9)	Midface (66.3%), Zygomatic complex (60.9%)	N/A	The majority of crashes occurred at night. Dysfunctional streetlight with no functional streetlight. Drivers and pillion affected. Associated alcohol influence
Peters (2017)	Journal article	Cohort (54)	Mean age = 30	General (9.9)	Mandible (34.6)	Zygomatic (9.9) and frontal bone (9.9)	N/A	Alcohol use at the time of injury. The majority of victims had delayed treatment that resulted in complications (Trismus)
Elm et al. (2014)	Journal article	Cross-sectional (42)	Mean age = 30.1	Laceration (37.8)	Mandible (64.4)	Nasal bone (24.4)	N/A	This call for the need to reduce violence and such traumas by improving the current socioeconomic and educational status
Stanford-Moore et al. (2022)	Dissertation	Retrospective (1203)	21–30 (41)	General (87.5). Laceration = 62.2	Mandible (56), the body of the mandible was 32.9% of the total mandibular fracture	Midface (32), Dentoalveolar made up 36.3% of the total midface fracture, followed by the zygomatic fracture of 32.5%	N/A	Associated injury =the head (60.8%) and limb (15%)
Stanslaus (2017)	Journal article	Cross-sectional (101)	20–29 (44.6)	Generalized. Abrasion (40.1)	Zygomatic complex (46)	Maxillary (28)	N/A	Incidence of 6.1%

### The sex distribution of participants

A total of 7317 participants were included, and of notable significance was that males were more affected than females. The male population was 5802 (79.3%), and 1515 women (20.7%) had a male-to-female ratio of 3.8:1. This scoping review revealed that sex significantly predicts maxillofacial injury among adults in SSA. Although the male-to-female ratio differs from one study to another, the included studies from East Africa accounted for the highest male-to-female ratio at 37.7:1 (Sohal et al. 2019) and 17:1 (Stanford-Moore et al. 2022), as indicated in Table 2. In contrast, studies from West Africa (Cameroon and Nigeria) recorded the lowest male-to-female disparity at 1.8:1 (Agbor et al. 2014) and 2:1 (Nwashindi et al. 2015), respectively, while the study in South Africa registered a ratio of 4.5:1 (Mogajane and Mabongo 2018).

### The peak age range of incidence

The peak age range of incidence for the included studies was between 18 and 40 years of age. However, this parameter varied from one author to another, and the most common incidence was between the age ranges of 21–30 years (Akhiwu et al. 2015; Tugaineyo 2011; Agbor et al. 2014; Chalya et al. 2011; Mpiima et al. 2018; Oginni et al. 2016; Nyameino et al. 2018; Kileo 2012; Kamulegeya et al. 2009; Teshome et al. 2017; Shah et al. 2014), representing 40.7% of the studies included in the review. Another peak of incidence age range employed by the authors was 20–29 years (Tsakiris et al. 2002; Obimakinde et al. 2018; Kiprop 2019; Udeabor et al. 2012; Agbara et al. 2018), representing 18.5% of the included articles. However, this information was unavailable in three studies (Table 3).

### Aetiological factors

The study identified various causes of MI as; RTC (Agbara et al. 2021; Agbor et al. 2014; Moshy et al. 2020), Assault (Tsakiris et al. 2002; Mogajane and Mabongo 2018; Mpiima et al. 2018), IPV (Pillay et al. 2018; Kiprop 2019), sport (Tekin and Ali 2021; Nwashindi et al. 2015), fall (Majambo et al. 2013), and domestic violence (Majambo et al. 2013), as evident in Table 2. The weighted percentage of RTC in the review accounted for 60.2% of the major causes of MI; however, motorcycle collisions constituted approximately 59.4% of the total RTC. The second most common cause of MI was assault/interpersonal violence (28.5 %). In contrast, studies from South Africa have identified IPV/Assault (Pillay et al. 2018; Tsakiris et al. 2002; Mogajane and Mabongo 2018) as the Major cause of MI at 55% (Pillay et al. 2018) and 60.3% (Mogajane and Mabongo 2018). The same observation was made in a study of the conflict-torn region (Tekin and Ali 2021). A significant number of studies have reported that the occurrence of MI is higher at night (Mogajane and Mabongo 2018; Chalya et al. 2011; Mpiima et al. 2018; Kiprop 2019; Nyameino et al. 2018; Moshy et al. 2020; Stanslaus 2017) and in urban residences (Agbor et al. 2014; Mpiima et al. 2018; Sohal et al. 2019; Kiprop 2019; Kileo 2012). Furthermore, the influence of alcohol and substance use were major contributing factors to MI (Stanford-Moore et al. 2022; Chalya et al. 2011; Mpiima et al. 2018; Sohal et al. 2019; Bernard et al. 2012; Kiprop 2019; Nyameino et al. 2018; Moshy et al. 2020; Teshome et al. 2017).

### Distribution of affected tissues

Sixteen studies (53.3%) of the included publications reported soft tissue injury (STI) of the maxillofacial area (Agbara et al. 2021; Pillay et al. 2018; Stanford-Moore et al. 2022; Tekin and Ali 2021; Tugaineyo 2011; Agbor et al. 2014; Chalya et al. 2011; Sohal et al. 2019; Bernard et al. 2012; Nyameino et al. 2018; Kileo 2012; Stanslaus 2017; Majambo et al. 2013; Udeabor et al. 2012; Agbara et al. 2018; Teshome et al. 2017), as indicated in Table 3. Facial soft injuries were reported by approximately 80% of the participants. However, a limited number of studies have reported the specific site (Agbara et al. 2021; Sohal et al. 2019; Nyameino et al. 2018; Majambo et al. 2013; Udeabor et al. 2012) and type of STI (Pillay et al. 2018; Tekin and Ali 2021; Kileo 2012; Udeabor et al. 2012; Teshome et al. 2017). The most common sites reported were the lips (Agbara et al. 2021; Nyameino et al. 2018; Majambo et al. 2013) and the frontal (Agbara et al. 2021; Agbara et al. 2018), while the orbit and cheek (Sohal et al. 2019) were reported in only one journal each. The most common STIs are lacerations (Pillay et al. 2018; Tekin and Ali 2021; Kileo 2012; Teshome et al. 2017) and abrasions (Udeabor et al. 2012; Teshome et al. 2017).

The review revealed that the mandible was the most frequently involved hard tissue (bone) in MI (Table 3), with an average weight of 56.6%. The middle third of the face (midface) was 27.8%. Commonly identified fracture sites in the mandible include the body (Obimakinde et al. 2018; Obimakinde et al. 2017; Kiprop 2019; Nyameino et al. 2018), symphysis (Pillay et al. 2018; Nwashindi et al. 2015), and parasymphyseal (Pillay et al. 2018; Nyameino et al. 2018; Nwashindi et al. 2015) regions. The identified regions on the midface are the maxilla (Tsakiris et al. 2002; Obimakinde et al. 2018; Agbor et al. 2014; Obimakinde et al. 2017; Krishnan et al. 2017; Kiprop 2019; Nyameino et al. 2018), zygoma (Agbara et al. 2021; Pillay et al. 2018; Stanford-Moore et al. 2022; Obimakinde et al. 2018; Kuye and Olufemi 2022; Sohal et al. 2019; Nwashindi et al. 2015), and nasal bone (Tekin and Ali 2021; Chalya et al. 2011). Frontal (Krishnan et al. 2017) and orbital (Nyameino et al. 2018) bones were identified in the upper third of the face. Two studies, Rwanda (Majambo et al. 2013) and Nairobi (Tugaineyo 2011), identified the involvement of dentoalveolar fractures at 59.3% and 24%, respectively.

### Burden of disease

The measures of morbidity frequency reported were the incidence and prevalence, and these were explored by four studies from the included articles (Pillay et al. 2018; Bernard et al. 2012; Majambo et al. 2013; Udeabor et al. 2012). The incidence rate of maxillofacial injury in a study from Kenya was 32.7% for the four-month period from September to December 2009 (Bernard et al. 2012). However, the annual incidence rate reported in a study conducted in Nigeria was 6.1% in 2008 (Udeabor et al. 2012). The prevalence of oral and maxillofacial injuries recorded among patients in Kigali Teaching Hospital was 16% as of June 2011 (Majambo et al. 2013), whereas that recorded among attending patients at the hospital in the Eastern Cape was 2.89% as of March 2016 (Pillay et al. 2018). The review further revealed that head and long bone injuries were the most associated injuries sustained with MI (Tugaineyo 2011; Obimakinde et al. 2018; Chalya et al. 2011; Oginni et al. 2016; Obimakinde et al. 2017; Bernard et al. 2012; Kiprop 2019; Kamulegeya et al. 2009; Agbara et al. 2018; Teshome et al. 2017). The associated head injury resulted in an altered level of consciousness with a Glasgow coma scale (GCS) score of 15 and below (Chalya et al. 2011; Oginni et al. 2016; Obimakinde et al. 2017; Agbara et al. 2018). The injury severity scale (ISS) was recorded in only one article (Chalya et al. 2011), where 37.5% of the patients had an ISS of ≥ 16. Two studies reported mortality due to maxillofacial injury (Tsakiris et al. 2002; Chalya et al. 2011). A study from the Accident and Emergency Department in Tanzanian Hospital reported a mortality rate of 11.7% among patients admitted between November 2008 and October 2009 (Chalya et al. 2011). Furthermore, another study from three academic hospitals in South Africa reported a significant association between abnormal airway status and the death of admitted patients after gunshot wounds (Tsakiris et al. 2002).

### Financial burden

Three articles (Akhiwu et al. 2015; Famurewa et al. 2021; Agbor et al. 2014), representing 11.1% of the included publications, reported the costs of managing MI. Studies from Kano (Akhiwu et al. 2015) and Ile-Ife (Famurewa et al. 2021) in Nigeria reported on the management of mandibular fractures, whereas those from Cameroon (Agbor et al. 2014) were on dentofacial fractures. The cost of management ranged from $200–$468.6. Most of the payment was made out of the patient’s pocket and was perceived to be expensive (Agbor et al. 2014).

## Discussion

The findings in this review reported varied evidence regarding the epidemiology and financial burden of MI in SSA. The knowledge gained from this review is crucial for preventing injury, with special attention to the facial region. It helps in developing targeted preventive measures, improving trauma-care protocols and allocating appropriate resources for the management and treatment of MI. The information acquired is critical for informing health strategies that are significant to policies and interventional efforts.

The review revealed that sex significantly predicted MI among adults in SSA. Similarly, studies have corroborated that male has a strong preponderance for MI (Juncar et al. 2021; Abosadegh and Rahman 2018; Alqahtani et al. 2020; Khan et al. 2022; Abhinav et al. 2019). The higher proportion of males has been ascribed to their direct involvement in social, economic, and cultural activities, resulting in them being more susceptible to traffic accidents, violence, and sports accidents (Abosadegh and Rahman 2018; Marsicano et al. 2019). In contrast, a multicentre study conducted in Europe on the elderly (Brucoli et al. 2020) revealed a higher female representation. Though, this was attributable to the higher female life expectancy in most European countries from 2001 to 2016. Furthermore, studies indicate that victims of domestic violence, often held back by financial or emotional ties, go unreported, making it a challenge to identify victims, thereby distorting the actual male-to-female ratios (Mayrink et al. 2020; Costa et al. 2014; Nóbrega et al. 2017). In this review, the observed wide difference in male-to-female ratios between East and West African studies may be due to cultural and social factors. In Tanzania, women rarely ride motorcycles and encourage cautious driving, which leads to a safer riding style (Sohal et al. 2019). Our findings further revealed that the peak age range of incidence is between 18 and 40 years, which is consistent with several studies (Saperi et al. 2017; Juncar et al. 2021; Manodh et al. 2016; Xiao-Dong et al. 2020; Conceição et al. 2018). Studies have revealed that people within this age range are more physically, professionally, and socially active (Juncar et al. 2021; Țenț et al. 2019), which may result in the practice of dangerous exercises, sports, carelessly driving motor vehicles, and engaging in outdoor activities that predispose them to trauma (Abosadegh and Rahman 2018; Al-Bokhamseen and Al-Bodbaij 2019; Abosadegh et al. 2017). Consequently, gender-specific prevention strategies targeting this age group may be effective in ameliorating the burden of MI.

The review provides valuable insights into the causes, distribution, and characteristics of (MI). Road traffic collisions (RTC) emerged as the primary cause, which is consistent with studies conducted in LMICs (Khan et al. 2022; Abhinav et al. 2019) and upper-medium-income countries (UMICs) (Ribeiro et al. 2016; Abosadegh et al. 2017). This review highlights that MI from road traffic collisions can be more frequent in areas with poor traffic laws, high congestion, reckless driving, and driver negligence. Motorcycle collisions account for a substantial proportion of RTC-related injuries in the East and West African countries, where motorcycles are used for commercial purposes. In line with this, studies have now identified motorcycle crashes as a significant threat to the heads, limbs, and lives of vulnerable road users` in developing countries (Boonkasem et al. 2015; Adeleye et al. 2019). Alcohol/illicit drug use, poor knowledge of traffic regulations, more than one “pillion” rider, lack of rider license, non-observance of traffic regulations, and non-use of helmets have been associated with motorcycle collisions. Interestingly, the occurrence of MI was found to be more prevalent at night and among urban residents, suggesting potential risk factors associated with these contexts. Soft tissue injuries, particularly to the lips, were prevalent among the participants, while the mandible was the most fractured hard tissue, followed by the midface region involving the maxilla, zygoma, and nasal bones. Research indicates that the mandible is prone to fractures due to its prominence, mobility, and susceptibility to violence. Despite its strength, it has weak points, rendering it more fracture-prone than midfacial bones (Mogajane and Mabongo 2018; Chalya et al. 2011; Kamulegeya et al. 2009). The elevated occurrence of dentoalveolar fractures in Nairobi compared with Rwanda may result from the frequent use of motorcycles in Rwanda. The helmet protects most parts of the head but leaves the dentoalveolar region less protected. Our review found geographic variations in incidence/prevalence due to urbanization, socioeconomic status, culture, crime, period, and environment (Pillay et al. 2018; Bernard et al. 2012; Majambo et al. 2013; Udeabor et al. 2012). However, the high incidence of MI observed in the Kenyan study (Bernard et al. 2012) may be attributed to the data collection period. Studies have revealed that RTCs tend to increase during the festive period because of heightened economic activity and potentially greater alcohol consumption (Agbor et al. 2014; Lerdsuwansri 2022). The measures of morbidity frequency in this study were considerable, further emphasizing the need for injury prevention. This review emphasizes the association between MI and head injury, which is demonstrated by loss of consciousness. The GCS score, which defines the extent of impaired consciousness, was as low as 3 (Chalya et al. 2011; Oginni et al. 2016; Obimakinde et al. 2017) while the ISS, which measures the level of injury severity, was greater than 16. The GCS and ISS aligned with the study conducted in Qatar (Al-Hassani 2019) where the mean GCS and ISS were 11.6 and 17.6, respectively. However, adopting an appropriate helmet/gear (Obimakinde et al. 2018; Obimakinde et al. 2017; Krishnan et al. 2017; Moshy et al. 2020), and following speed-limit regulations (Moshy et al. 2020) significantly reduces head injury severity in commercial motorcyclists. Importantly, the involvement of compromised airways has been reported (Tsakiris et al. 2002; Stanslaus 2017), which may result in the death of the victim (Tsakiris et al. 2002; Chalya et al. 2011).

Several studies have explored facial injury epidemiology and management; however, cost information has frequently not been reported (Saperi et al. 2017). The financial burden of managing facial injuries includes direct and indirect costs stemming from the rehabilitation and restoration of aesthetic, physical, and functional damage (Conceição et al. 2018; Altiparmak et al. 2020). In this review, the direct cost was reported by two included studies (Famurewa et al. 2021; Agbor et al. 2014), while only one study (Akhiwu et al. 2015) reported the cost of illness (direct and indirect costs due to days of lost productivity)) for the mandibular fractures. Our study found mandibular fracture management costs ranging from $200 to $468.6, notably lower than the US average hospital cost of $26,000 for closed reduction procedures (Nalliah et al. 2013). This cost disparity can be attributed to the higher US healthcare costs, resulting in the high cost of facial fracture management (Nalliah et al. 2013). In 2008, the total hospitalization charges for facial fracture reduction in the USA amounted to $1.06 billion (Nalliah et al. 2013). In contrast, the simpler healthcare system and the lower costs emanating from the use of non-proprietary plating systems, and outpatient settings with local anaesthesia (reducing operating room and general anaesthesia fees) result in a lower financial burden in SSA. (Famurewa et al. 2021). In Malaysia, government subsidies for public hospitals are substantial (Saperi et al. 2017), as obtained in most SSA countries. A study in Nigeria found that treating mandibular fractures accounted for 8.4% of the state’s healthcare budget, equivalent to 15.2% of the 2015 GDP per capita (Akhiwu et al. 2015). However, patients in SSA countries still perceive expenses to be costly because of the lack of accessible Insurance and Social protection schemes. As a result, individuals and their families bear the financial burden (Out-of-pocket) (Famurewa et al. 2021; Sangowawa et al. 2011), potentially leading to further impoverishment of injured victims (Famurewa et al. 2021; Agbor et al. 2014). Addressing these disparities and improving access to affordable healthcare and social protection could alleviate the financial strain faced by victims and their families in SSA.

### Strengths and limitations

To our knowledge, this is the first scoping review to map evidence on the epidemiology of MI and its financial burden in SSA. A comprehensive search strategy identified numerous relevant studies using various designs. The review followed PRISMA guidelines and employed STROBE for reporting strength. However, the limitation of the search being limited to English publications may have led to the omission of other relevant articles.

## Conclusions

This review identified road traffic collisions and assaults as the primary causes of maxillofacial injury, with motorcycle collisions being prominent in areas where motorcycles are a major mode of transportation. Maxillofacial injury, when combined with a head injury, can be life-threatening, necessitating continuous advocacy for preventive measures and strict traffic rule enforcement. Likewise, targeted male-focused programs on substance abuse, anger management, and conflict resolution can further reduce the incidence of facial fractures resulting from assaults. Additional research is required to assess the costs associated with managing and rehabilitating MI as there is limited literature on this aspect. This information can inform resource allocation, policy development, advocacy efforts, and planning for injury prevention and management.

### Supplementary Information

**Additional file 1.** PRISMA-ScR Checklist.

**Additional file 2.** DATA BASE- search.

**Additional file 3.** Extraction form.

**Additional file 4.** STROBE Score for included studies.

## Data Availability

Available in tables or as supplementary material; there are no data repositories.

## Abbreviations

GCS: Glasgow coma scale
LIMCs: Low- and middle-income countries
MeSH: Medical subject headings
MI: Maxillofacial Injury
PCC: Population, concept, and content
PRISMA: Preferred reporting items for systematic reviews and meta-analysis
PRISMA-ScR: Preferred reporting items for systematic review and meta-analysis extension for scoping reviews
RTC: Road traffic collision
SSA: Sub-Saharan Africa
STROBE: Strengthening the reporting of observational studies in epidemiology
UMICs: Upper-medium-income countries
