# Supplementary material for: Epidemiology of maxillofacial injury among adults in sub-Saharan Africa: a scoping review
Source: Inj Epidemiol. 2023 Nov 15;10:58. doi: 10.1186/s40621-023-00470-5 (PMC10652446; doi:10.1186/s40621-023-00470-5)
Supplement: Supplementary file 2 — Additional file 2. DATA BASE- search. [file 40621_2023_470_MOESM2_ESM.docx]

**Additional File 2- DATA BASE SEARCH**

| **DATA BASE SEARCH** | | | | | |
| --- | --- | --- | --- | --- | --- |
| Population | Concept | Context | Key Words | Date | Number found |
| **SCOPUS** | | | | | |
| Adult, 18 years  and above with  maxillofacial  injury | Maxillofacial injury | Countries in sub-Saharan Africa | Facial  AND  injury  OR  maxillofacial  AND  trauma  OR  maxillofacial  AND  injury  OR  maxillary  AND  fracture  OR  maxillary  AND  injury  OR  mandibular  AND  fracture  OR  mandibular  AND  injury  OR  zygomatic  AND  fracture  OR  zygomatic  AND  injury  OR  nasal  AND  injury  AND  epidemiology  OR  incidence  OR  prevalence  OR  risk  AND  factors  OR  burden  OR  disability  OR  cost  AND NOT  children  AND NOT  cancer | 15/06/2022 | 2369 |
| **GOOGLE SCHOLAR** | | | | | |
| Adult, 18 years  and above with  maxillofacial  injury | Maxillofacial injury | Countries in sub-Saharan Africa | Facial injury, maxillofacial trauma, maxillofacial injury, maxillary fracture, maxillary injury, mandibular fracture, mandibular injury, zygomatic fracture, zygomatic injury, nasal injury, epidemiology, incidence, prevalence, risk factors, burden, disability, cost. | 01/08/2022 | 209 |
| **MEDLINE** | | | | | |
| Adult, 18 years  and above with  maxillofacial  injury | Maxillofacial injury | Countries in sub-Saharan Africa | facial injury AND epidemiology AND Costs | 05/08/2022 | 56 |
| **CINAHL** | | | | | |
| Adult, 18 years  and above with  maxillofacial  injury | Maxillofacial injury | Countries in sub-Saharan Africa | Maxillofacial injury, epidemiology, Costs, Africa | 17/08/2022 | 3406 |
| **PUBMED** | | | | | |
| Adult, 18 years  and above with  maxillofacial  injury | Maxillofacial injury | Countries in sub-Saharan Africa | ((("face"[MeSH Terms] OR "face"[All Fields] OR "facial"[All Fields] OR "facials"[All Fields]) AND ("injuries"[MeSH Subheading] OR "injuries"[All Fields] OR "trauma"[All Fields] OR "wounds and injuries"[MeSH Terms] OR ("wounds"[All Fields] AND "injuries"[All Fields]) OR "wounds and injuries"[All Fields] OR "trauma s"[All Fields] OR "traumas"[All Fields])) OR "maxillofacial injuries"[MeSH Terms] OR ("dentoalveolar"[All Fields] AND "fractures, bone"[MeSH Terms]) OR "epidemiology"[MeSH Terms]) AND ("economics"[MeSH Subheading] OR "economics"[All Fields] OR "cost"[All Fields] OR "costs and cost analysis"[MeSH Terms] OR ("costs"[All Fields] AND "cost"[All Fields] AND "analysis"[All Fields]) OR "costs and cost analysis"[All Fields]) | 10/08/2022 | 1996 |
| ScienceDirect | | | | | |
| Adult, 18 years  and above with  maxillofacial  injury | Maxillofacial injury | Countries in sub-Saharan Africa | Maxillofacial injury, epidemiology, Costs, Africa | 26/08/2022 | 202 |
| **Total = 8238** | | | | | |
| **Others** | | | | | |
| From references of included articles= 8 | | | | | |
