# Supplementary material for: Epidemiology of maxillofacial injury among adults in sub-Saharan Africa: a scoping review
Source: Inj Epidemiol. 2023 Nov 15;10:58. doi: 10.1186/s40621-023-00470-5 (PMC10652446; doi:10.1186/s40621-023-00470-5)
Supplement: Supplementary file 3 — Additional file 3. Extraction form. [file 40621_2023_470_MOESM3_ESM.pdf]

Mapping Evidence on the Epidemiology and Cost Associated with Maxillofacial Injury among Adults in Sub-Saharan Africa: A Scoping Review Protocol

Data charting (extraction) Form

* Indicates required question

1. Author & Date (Reference number)

2. Study setting (Country)

3. Publication type

4. Study design (Sample size)

5. Peak age range of incidence, year (%)

6. Male/Female ratio

7. Major cause of maxillofacial injury

8. 2nd Major cause of maxillofacial injury

9. Percentage Interpersonal violence related (%)

10. Maxillofacial soft tissue affected (%)

11. Most maxillofacial bone affected (%)

12. 2nd Most Maxillofacial bone affected (%)

13. Cost of Management ($)

14. Relevant findings

15. Conclusions

16. Screener's initial *

____

This content is neither created nor endorsed by Google.

Google Forms
