# Supplementary material for: Epidemiology of maxillofacial injury among adults in sub-Saharan Africa: a scoping review
Source: Inj Epidemiol. 2023 Nov 15;10:58. doi: 10.1186/s40621-023-00470-5 (PMC10652446; doi:10.1186/s40621-023-00470-5)
Supplement: Supplementary file 4 — Additional file 4. STROBE Score for included studies. [file 40621_2023_470_MOESM4_ESM.docx]

**Additional File 4: Strengthening the reporting of observational studies in epidemiology (STROBE) for included articles.**

| Selected articles & years | | ITEMS | | | | | | | | | | | | | | | | | | | | | | Total |
| --- | --- | --- | --- | --- | --- | --- | --- | --- | --- | --- | --- | --- | --- | --- | --- | --- | --- | --- | --- | --- | --- | --- | --- | --- |
|  |  | 1 | 2 | 3 | 4 | 5 | 6 | 7 | 8 | 9 | 10 | 11 | 12 | 13 | 14 | 15 | 16 | 17 | 18 | 19 | 20 | 21 | 22 |  |
| R. Agbara, A. E. Obiechina, S. O. Ajike and D. S. Adeola (2018) | | 1 | 1 | 1 | 1 | 1 | 1 | 1 | 1 | - | 1 | 1 | 1 | 1 | 1 | 1 | 1 | 1 | 1 | 1 | 1 | 1 | - | 20 |
| R. Agbara, B. Fomete and K. U. Omeje (2021) | | 1 | 1 | - | 1 | 1 | 1 | 1 | 1 | 1 | 1 | 1 | 1 | 1 | 1 | 1 | 1 | 1 | - | 1 | 1 | 1 | 1 | 20 |
| A. Agbor, C. Azodo, E. B. Ebot and S. Naidoo (2014) | | 1 | 1 | - | 1 | 1 | 1 | 1 | 1 | - | 1 | 1 | - | 1 | 1 | 1 | 1 | 1 | - | 1 | 1 | 1 | - | 17 |
| B. Akhiwu, H. Suleiman, M. Muktar & I. Amole (2015) | | 1 | 2 | - | 1 | 1 | 1 | 1 | 1 | - | - | 1 | - | 1 | 1 | 1 | 1 | - | - | - | 1 | - | - | 13 |
| E. Bernard, M. K. Akama, W. Odhiambo, M. Chindia and B. Mua (2012) | | 1 | 1 | 1 | 1 | 1 | 1 | 1 | 1 | - | - | 1 | 1 | 1 | 1 | 1 | 1 | - | 1 | - | 1 | - | - | 16 |
| P. L. Chalya, M. McHembe, J. B. Mabula, E. S. Kanumba and J. M. Gilyoma (2011) | | 1 | 1 | - | 1 | 1 | 1 | 1 | 1 | - | - | 1 | 1 | 1 | 1 | 1 | 1 | - | - | - | 1 | - | 1 | 15 |
| J. Kiprop (2019) | | 1 | 1 | 1 | 1 | 1 | 1 | 1 | 1 | 1 | 1 | 1 | 1 | 1 | 1 | 1 | 1 | 1 | 1 | 1 | 1 | 1 | - | 21 |
| B. A. Famurewa, S. B. Aregbesola, O. T. Alade and T. A. Akinniyi (2021) | | 1 | 1 | - | 1 | 1 | 1 | 1 | 1 | - | - | 1 | 1 | 1 | 1 | 1 | 1 | 1 | 1 | 1 | 1 | 1 | - | 18 |
| A. Kamulegeya, F. Lakor and K. Kabenge (2009) | | 1 | 1 | 1 | 1 | 1 | 1 | 1 | 1 | - | 1 | 1 | 1 | 1 | 1 | 1 | 1 | 1 | 1 | - | 1 | 1 | - | 19 |
| B. F. Kileo (2012) | | 1 | 1 | 1 | 1 | 1 | 1 | 1 | 1 | - | 1 | 1 | 1 | 1 | 1 | 1 | 1 | 1 | 1 | 1 | 1 | 1 | - | 20 |
| U. Krishnan, R. Byanyima, A. Faith and A. Kamulegeya (2017) | | 1 | 1 | - | 1 | 1 | 1 | 1 | 1 | - | 1 | 1 | 1 | 1 | 1 | 1 | 1 | 1 | 1 | - | - | 1 | 1 | 18 |
| O. F. Kuye and O. Olufemi (2022) | | 1 | 1 | 1 | 1 | 1 | 1 | 1 | 1 | - | - | 1 | 1 | 1 | 1 | 1 | 1 | 1 | 1 | 1 | 1 | - | 1 | 19 |
| M. Majambo, R. Sasi, C. Mumena, G. Museminari, J. Nzamukosha, A. Nzeyimana, et al. (2013) | | 1 | 1 | 1 | 1 | 1 | 1 | 1 | 1 | - | - | 1 | 1 | 1 | 1 | 1 | 1 | - | 1 | - | 1 | 1 | - | 17 |
| B. Mogajane and M. Mabongo (2018) | | 1 | 1 | 1 | 1 | 1 | 1 | 1 | 1 | - | - | 1 | 1 | 1 | 1 | 1 | 1 | 1 | 1 | - | 1 | - | - | 17 |
| J. R. Moshy, B. S. Msemakweli, S. S. Owibingire and K. S. Sohal (2020) | | 1 | 1 | 1 | 1 | 1 | 1 | 1 | 1 | - | - | 1 | 1 | 1 | 1 | 1 | 1 | 1 | 1 | - | 1 | - | - | 17 |
| P. Mpiima, A. Kasangaki, E. Nkamba and C. M. Rwenyonyi (2018) | | 1 | 1 | 1 | 1 | 1 | 1 | 1 | 1 | - | 1 | 1 | 1 | 1 | 1 | 1 | 1 | 1 | 1 | - | 1 | - | - | 18 |
| A. Nwashindi, E. M. Dim, F. U. Uduma and B. B. Akhiwu (2015) | | 1 | 1 | - | 1 | 1 | 1 | 1 | 1 | - | 1 | 1 | 1 | 1 | 1 | 1 | 1 | - | - | - | 1 | 1 | - | 16 |
| J. Nyameino (2016) | | 1 | 1 | - | 1 | 1 | 1 | 1 | 1 | - | - | 1 | 1 | 1 | 1 | 1 | 1 | 1 | - | 1 | 1 | - | - | 16 |
| O. S. Obimakinde, K. O. Ogundipe, T. B. Rabiu and V. N. Okoje (2017) | | 1 | 1 | 1 | 1 | 1 | 1 | 1 | 1 | - | 1 | 1 | 1 | 1 | 1 | 1 | 1 | 1 | 1 | - | 1 | - | - | 18 |
|  | | 1 | 2 | 3 | 4 | 5 | 6 | 7 | 8 | 9 | 10 | 11 | 12 | 13 | 14 | 15 | 16 | 17 | 18 | 19 | 20 | 21 | 22 |  |
| O. S. Obimakinde, O. A. Olajuyin, T. B. Rabiu and O. J. Olanrewaju (2018) | | 1 | 1 | 1 | 1 | 1 | 1 | 1 | 1 | - | 1 | 1 | 1 | 1 | 1 | 1 | 1 | 1 | 1 | - | 1 | - | 1 | 19 |
| F. O. Oginni, T. Oladejo, D. P. Alake, J. O. Oguntoba and O. F. Adebayo (2016) | | 1 | 1 | 1 | 1 | 1 | 1 | 1 | 1 | - | 1 | 1 | 1 | 1 | 1 | 1 | 1 | 1 | 1 | - | 1 | 1 | - | 19 |
| A. Teshome, G. Andualem, R. Tsegie and S. Seifu (2017) | | 1 | 1 | 1 | 1 | 1 | 1 | 1 | 1 | - | 1 | 1 | 1 | 1 | 1 | 1 | 1 | 1 | 1 | - | 1 | 1 | - | 20 |
| P. Tsakiris, P. E. Cleaton-Jones and M. A. Lownie (2002) | | 1 | 1 | - | 1 | 1 | 1 | 1 | 1 | - | 1 | 1 | 1 | 1 | 1 | 1 | 1 | 1 | - | - | 1 | 1 | - | 17 |
| L. Pillay, M. Mabongo and B. Buch (2018) | | 1 | 1 | - | 1 | 1 | 1 | 1 | 1 | - | 1 | 1 | 1 | 1 | 1 | 1 | 1 | 1 | - | - | 1 | - | - | 16 |
| K. S. Sohal, B. M. Kalyanyama and S. S. Owibingire  (2019) | | 1 | 1 | - | 1 | 1 | 1 | 1 | 1 | - | - | 1 | 1 | 1 | 1 | 1 | 1 | 1 | - | - | 1 | - | - | 15 |
| G. B. Stanford-Moore, G. Niyigaba, G. Tuyishimire, J. Yau, A. Kulkrani, V. Nyabyenda, et al. (2022) | | 1 | 1 | 1 | 1 | 1 | 1 | 1 | 1 | - | - | 1 | 1 | 1 | 1 | 1 | 1 | 1 | 1 | - | 1 | - | 1 | 18 |
| A. M. Tekin and I. M. Ali (2021) | | 1 | 1 | - | - | 1 | 1 | 1 | 1 | - | - | 1 | 1 | 1 | 1 | 1 | 1 | 1 | - | - | 1 | - | - | 14 |
| B. Stanslaus (2017) | | 1 | 1 | 1 | 1 | 1 | 1 | 1 | 1 | - | 1 | 1 | 1 | 1 | 1 | 1 | 1 | 1 | 1 | 1 | 1 | 1 | - | 20 |
| E. I. Tugaineyo (2011) | | 1 | 1 | 1 | 1 | 1 | 1 | 1 | 1 | - | 1 | 1 | 1 | 1 | 1 | 1 | 1 | 1 | 1 | 1 | 1 | 1 | - | 20 |
| S. E. Udeabor, V. I. Akinmoladun, O. A. Fasola and A. E. Obiechina (2012) | | 1 | 1 | - | 1 | 1 | 1 | 1 | 1 | - | - | 1 | 1 | 1 | 1 | 1 | 1 | - | - | - | 1 | 1 | - | 15 |
| **Average STROBE Score = 17.6** | | | | | | | | | | | | | | | | | | | | | | | | |

Keys

| \| Item number \|  \|  \|  \| \| --- \| --- \| --- \| --- \| \| 1 \| Title and abstract \|  \|  \| \| Introduction \| \|  \|  \| \| 2 \| Background/Rationale \|  \|  \| \| 3 \| Objectives \|  \|  \| \| Methods \| \|  \|  \| \| 4 \| Study Design \|  \|  \| \| 5 \| Setting \|  \|  \| \| 6 \| Participants \|  \|  \| \| 7 \| Variables \|  \|  \| \| 8 \| Data sources/ Measurement \|  \|  \| \| 9 \| Bias \|  \|  \| \| 10 \| Study size \|  \|  \| \| 11 \| Quantitative variables \|  \|  \| \| 12 \| Statistical methods \|  \|  \| \| Results \| \|  \|  \| \| 13 \| Participants \|  \|  \| \| 14 \| Descriptive data \|  \|  \| \| 15 \| Outcome data \|  \|  \| \| 16 \| Main results \|  \|  \| \| 17 \| Other analysis \|  \|  \| \| Discussion \| \|  \|  \| \| 18 \| Key results with reference to objectives \|  \|  \| \| 19 \| Limitation \|  \|  \| \| 20 \| Interpretation \|  \|  \| \| 21 \| Generalizability \|  \|  \| \| Other information \| \|  \|  \| \| 22 \| Funding \|  \|  \| |
| --- | --- | --- | --- | --- | --- | --- | --- | --- | --- | --- | --- | --- | --- | --- | --- | --- | --- | --- | --- | --- | --- | --- | --- | --- | --- | --- | --- | --- | --- | --- | --- | --- | --- | --- | --- | --- | --- | --- | --- | --- | --- | --- | --- | --- | --- | --- | --- | --- | --- | --- | --- | --- | --- | --- | --- | --- | --- | --- | --- | --- | --- | --- | --- | --- | --- | --- | --- | --- | --- | --- | --- | --- | --- | --- | --- | --- | --- | --- | --- | --- | --- | --- | --- | --- | --- | --- | --- | --- | --- | --- | --- | --- | --- | --- | --- | --- | --- | --- | --- | --- | --- | --- | --- | --- | --- | --- | --- | --- | --- | --- | --- | --- |
